# Transumbilical single-site laparoscopic resection of splenic papillary angioendothelioma in a pediatric patient: a rare case report and review of literature

**DOI:** 10.3389/fonc.2025.1571209

**Published:** 2025-07-14

**Authors:** Meng Kong, Qingfei Zhai, Shisong Zhang, Hongzhen Liu, Yuexia Bai, Shuai Chen, Jinhua Jia, Dong Wang, Xiang Ma

**Affiliations:** ^1^ Department of Pediatric Surgery, Children’s Hospital Affiliated to Shandong University, Jinan, China; ^2^ Department of Pediatric Surgery, Jinan Children’s Hospital, Jinan, China; ^3^ Department of Ultrasound Medicine, Jining No.1 People’s Hospital, Jining, China; ^4^ Department of Pathology, Children’s Hospital Affiliated to Shandong University, Jinan, China; ^5^ Department of Pediatric Surgery, Shandong Provincial Maternal and Child Health Care Hospital Affiliated to Qingdao University, Jinan, China; ^6^ Department of Respiratory Disease, Children’s Hospital Affiliated to Shandong University, Jinan, China

**Keywords:** splenic papillary angioendothelioma, pediatric, laparoscopy, single-site, partial splenectomy

## Abstract

**Background:**

Splenic papillary angioendothelioma (PILA) is a rare borderline vascular tumor that is often asymptomatic and typically identified incidentally during imaging studies. Owing to its nonspecific clinical presentation and diagnostic complexity, optimal management strategies, particularly in pediatric patients, remain understudied. This report describes a case of pediatric splenic PILA managed with spleen-preserving minimally invasive surgery.

**Case description:**

A 5-year-old boy presented with an incidentally detected splenic mass during abdominal ultrasound six months prior to admission. The patient was asymptomatic and had no prior medical history. The patient was 115 cm tall and weighed 20.5 kg when admitted to the hospital. In the past half year, there has been no obvious growth and development delay, nor have there been any significant changes in diet or weight. Preoperative imaging, including contrast-enhanced ultrasound (US), computed tomography (CT), and magnetic resonance imaging (MRI), revealed a well-circumscribed lesion (6.0 cm × 5.5 cm × 5.0 cm) at the splenic upper pole, exhibiting heterogeneous enhancement patterns suggestive of a vascular tumor. Following multidisciplinary evaluation, a transumbilical single-port laparoscopic exploration was performed. Intraoperative frozen-section histopathology revealed benign characteristics, suggesting the need for a spleen-sparing partial splenectomy. Definitive postoperative histopathological and immunohistochemical analyses confirmed the diagnosis of PILA, which was characterized by CD31, CD34, and ERG positivity.

**Conclusions:**

Pediatric splenic PILA is diagnostically challenging because of its rarity and overlapping radiological features with other vascular neoplasms. Complete surgical excision and longitudinal surveillance are critical to mitigate the risk of local recurrence or metastasis, given the tumor’s borderline malignant potential. Single-port laparoscopic partial splenectomy represents a feasible, minimally invasive approach for pediatric patients, balancing oncological safety with preservation of splenic function. This case underscores the importance of integrating intraoperative frozen-section analysis and advanced imaging to guide surgical decision-making, particularly in children.

## 1 Introduction

Endovascular papillary angioendothelioma (EPA), also known as Dabska tumor and papillary intralymphatic angioendothelioma (PILA), is a rare borderline tumor that lies between hemangiomas and angiosarcomas. It was first reported by Maria Dabska in 1969 in six children (1). PILA most commonly occurs in the skin and subcutaneous tissues (2), with rare occurrences in the spleen, testes, tongue, and bones (3–6). Overall, PILA has a good prognosis, but there is a possibility of local recurrence and low-grade metastasis (7). Splenic PILA is clinically insidious, with most patients being asymptomatic, and it is usually discovered incidentally. However, it may cause abdominal discomfort, pain, or a palpable abdominal mass, and in very rare cases, abdominal bleeding, infection, or splenic rupture may occur (8). The diagnosis of splenic PILA cannot rely solely on clinical presentation and imaging; pathological examination remains the gold standard for diagnosis. Here, we report a case of pediatric splenic PILA, which was incidentally identified via abdominal ultrasound, and we performed partial splenectomy through single-port laparoscopic assistance, preserving normal splenic tissue and function while minimizing trauma, promoting rapid recovery, and achieving good cosmetic results (9). Through the discussion of this case, we aim to share valuable insights for diagnosing and treating splenic PILA.

## 2 Case report

### 2.1 General information

The patient was a 5-year-old male who was admitted because of “an incidental finding of a splenic mass during an abdominal ultrasound six months ago.” Six months prior, the child underwent a health check at a local hospital, where an abdominal ultrasound suggested a splenic lymphangioma. The physician recommended surgical treatment, but the parent opted for observation instead. During this period, the child remained in good general condition, with no fever, nausea, vomiting, abdominal pain, diarrhea, or other discomfort. He presented to our hospital for re-evaluation, where abdominal ultrasound raised a high suspicion of a splenic vascular tumor, which appeared slightly enlarged compared with previous findings. He was admitted with a diagnosis of a “splenic mass.” The child had no history of trauma, no known drug or food allergies, no history of living in a parasitic area, no family history of hereditary or infectious diseases, and no history of animal contact.

### 2.2 Auxiliary examination

**Figure 1:**
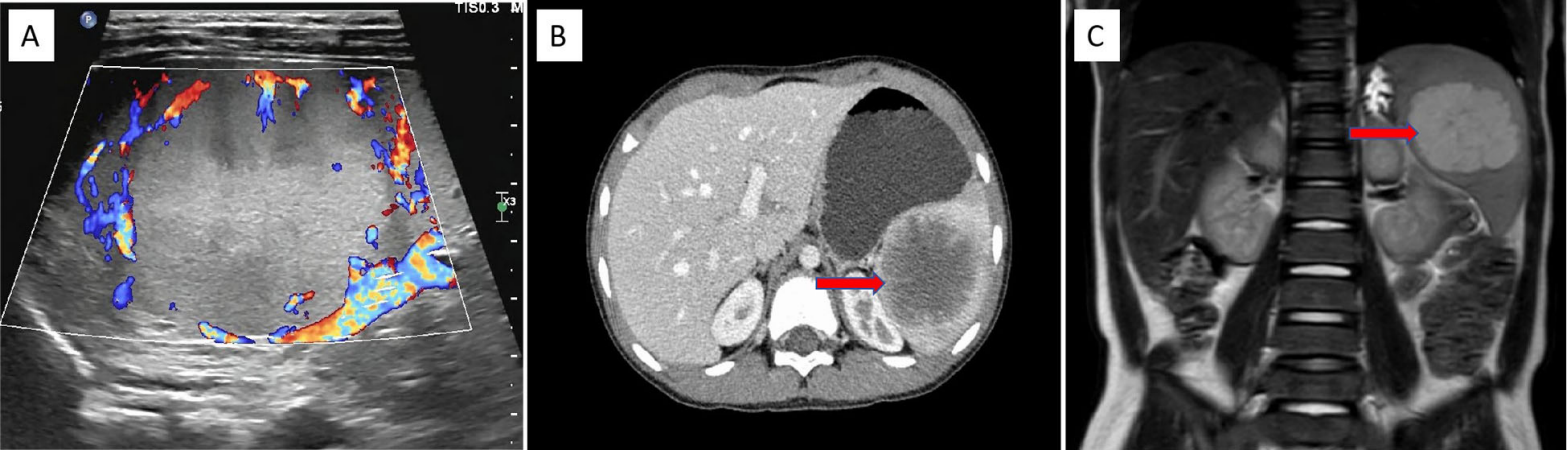
US, CT, and MRI findings of the splenic PILA. **(A)** Abdominal US image showing a medium echogenic area at the upper pole of the spleen, approximately 6.0 cm × 5.5 cm × 5.0 cm in size, with clear boundaries and abundant blood flow signals around the mass (coronal plane). **(B)** Abdominal CT image showing splenomegaly with a low-density mass visible within, approximately 6.3 cm × 5.7 cm × 5.2 cm in size, with relatively clear boundaries. After enhancement scanning, the edges and some internal parts of the lesion show mild to moderate enhancement (horizontal plane). This image displays a cross-section of the spleen. **(C)** Abdominal MRI image showing splenomegaly with clear boundaries, a slightly low signal in the center, and significant edge enhancement during the arterial phase of enhancement scanning. In the portal phase, there is progressive enhancement towards the center, resembling a spoke-like enhancement, with a signal higher than the splenic parenchyma. The central low signal shows no significant enhancement, and the lesion measures approximately 6.0 cm × 5.2 cm × 5.0 cm (coronal plane).

Physical examination revealed a flat abdomen with no visible abdominal wall varices, no gastrointestinal peristalsis or waves, a soft abdomen without tenderness, rebound tenderness, or muscle rigidity. The liver was not palpable below the costal margin, whereas the spleen was palpable 5 cm below the costal margin, with a cystic mass of approximately 6 cm × 5 cm in the left upper abdomen and poor mobility. Laboratory tests revealed a white blood cell count of 8.18 × 10^9^/L, red blood cell count of 4.62 × 10^12^/L, hemoglobin level of 123 g/L, and platelet count of 285 × 10^9^/L. The biochemical results revealed a total protein concentration of 65 g/L, globulin concentration of 21 g/L, albumin/globulin ratio of 2.09, total bilirubin concentration of 6.8 μmol/L, and direct bilirubin concentration of 4.5 μmol/L. Serum tumor marker levels were unremarkable. Abdominal ultrasonography revealed splenomegaly with an irregular shape and a moderately echoic mass measuring approximately 6.0 cm × 5.5 cm × 5.0 cm with clear boundaries and heterogeneous internal echoes surrounded by abundant blood flow signals (**Figure 1A**). CT scans revealed a well-defined, enlarged spleen with a low-density mass of approximately 6.3 cm × 5.7 cm × 5.2 cm, with some unclear borders, and enhancement scans revealed mild to moderate enhancement at the edges and within the lesion, which was consistent with a splenic mass, with a high suspicion of a vascular lesion (**Figure 1B**). MRI revealed a well-defined, enlarged spleen with a mass exhibiting long T1 and long T2 signals, with a central area of slightly shorter T1 and T2 signals, clear boundaries, and significant enhancement during the arterial phase, with progressive enhancement towards the center during the portal phase, resembling wheel-like enhancement. The maximum extent of the lesion was approximately 6.0 cm × 5.2 cm × 5.0 cm. The preliminary diagnosis was a possible sclerosing vascular lesion of the spleen (**Figure 1C**).

### 2.3 Surgical procedure

On the basis of the patient’s medical history, physical examination, and auxiliary tests, there is a high possibility of a vascular tumor in the spleen. On the premise of excluding surgical contraindications and after providing prepared suspended red blood cells before the operation, we performed the surgery on the patient on the fourth day after admission. Given that the child is only 5 years old, the diameter of the tumor is greater than 5 cm, and its location is at the upper pole of the spleen, excluding the contraindications for the operation, after giving suspended red blood cells before the operation, the operation was performed on the 4th day after admission. We opted for single-site laparoscopic surgery through the belly button. During the procedure, a small amount of tissue was removed and sent for rapid pathology, and the sample was preliminarily diagnosed as a benign tumor of the spleen. Therefore, we proceeded with partial splenectomy.

**Figure 2:**
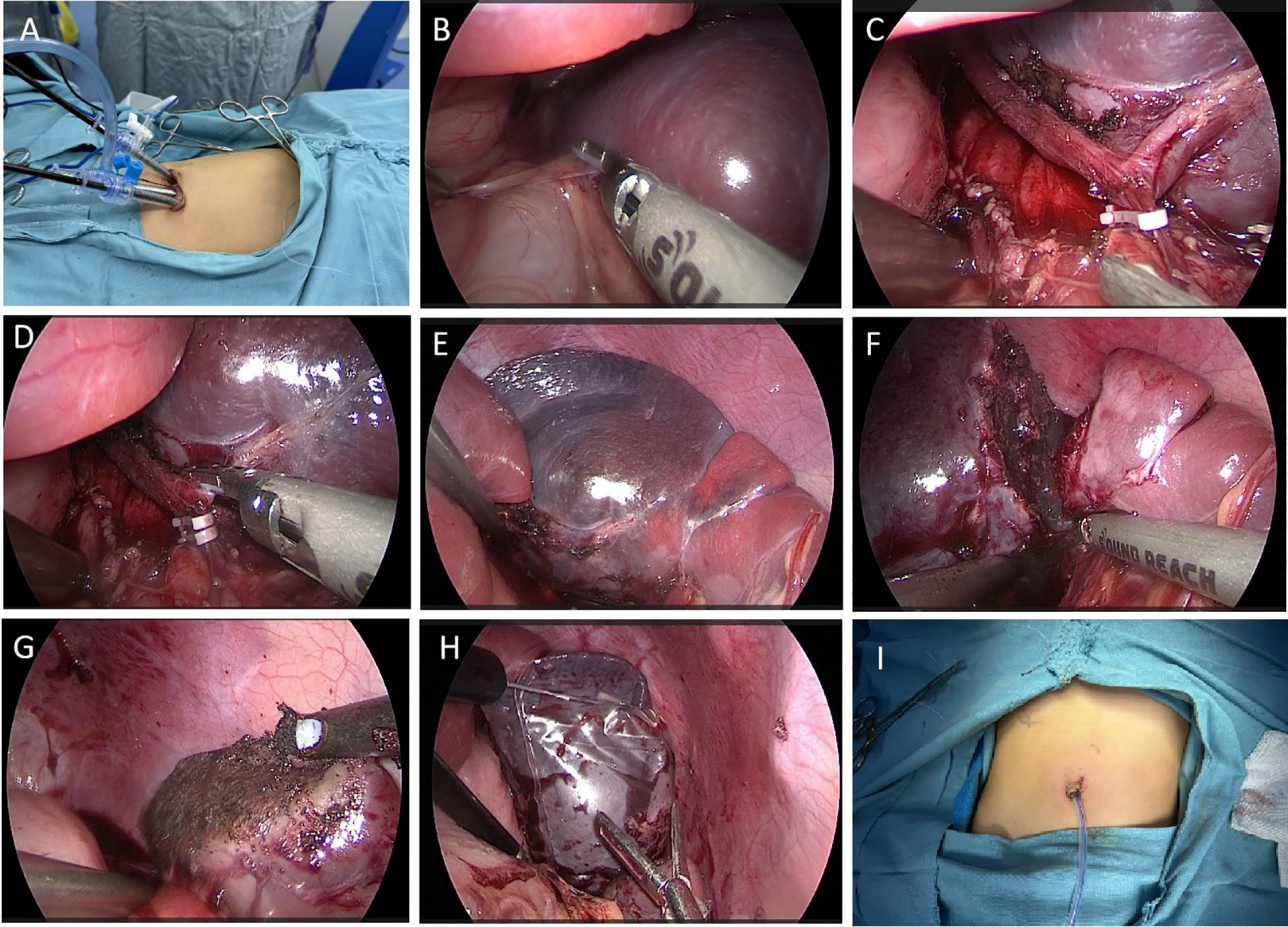
Surgical methods and steps. **(A)** The position of the trocars at the umbilicus. **(B)** LigaSure cuts the gastrocolic ligament, exposing the splenic hilum. **(C)** The branch vessels of the splenic lesion segment were dissected and occluded with Hem-o-lok clips. **(D)** LigaSure cuts the branch vessels. **(E)** A significant ischaemic line is visible at the upper pole of the spleen. **(F)** LigaSure was used to excise the diseased spleen along the ischemic line. **(G)** The splenic wound is cauterized for hemostasis with a microwave knife. **(H)** The excised spleen was placed into a specimen bag. **(I)** A drainage tube was placed at the umbilicus.

The operation was as follows: 1) After anaesthesia, the child was placed in a supine position with the legs spread approximately 45° apart, the head elevated and feet lowered, and the left flank elevated by approximately 30°. A gastric tube and urinary catheter were placed preoperatively. The laparoscope was positioned on the left shoulder of the child, with the surgeon located between the child’s legs and the assistant on the right side. A vertical incision of approximately 2 cm was made at the umbilicus, and the subcutaneous tissue was carefully separated from the muscle layer. 2) A 5 mm trocar was inserted at the center of the umbilicus to create CO_2_ gas in the abdomen at 8 mmHg pressure and a flow rate of 6 L/min, followed by the insertion of a 30° laparoscope. Two additional 5 mm trocars were placed on the left and right sides of the umbilicus as working ports (**Figure 2A**), and a needle was inserted approximately 3 cm to the right of the xiphoid process to suspend the greater curvature of the stomach near the fundus to the abdominal wall, maintaining appropriate tension. 3) The left gastrocolic ligament was first cut via LigaSure (**Figure 2B**), which revealed that the tumor was located in the upper part of the spleen. During the procedure, a small amount of splenic tissue was removed and sent for rapid pathology, which indicated a benign tumor but did not exclude a vascular origin. We then decided to perform a partial splenectomy, continuing to isolate the splenogastric and splenodiaphragmatic ligaments and ligating the short gastric vessels. 4) The splenic hilum capsule was opened, and the vascular branches of the affected segment were isolated along the splenic artery and vein. After the branch vessels were fully isolated, they were ligated and divided via Hem-o-lok clips (**Figures 2C, D**). 5) An obvious ischaemic line was observed on the spleen (**Figure 2E**), and the splenic lesion was excised from the outer side of the ischaemic line via LigaSure (**Figure 2F**), with good bleeding control via electrocautery on the cut surface (**Figure 2G**). 6) The excised spleen was placed in a specimen bag, and the three trocars at the umbilicus were removed (**Figure 2H**). The umbilical incision was opened and widened, and after the opening of the specimen bag was tightened, it was removed from the body. The specimen was fragmented using oval clamps and removed for pathology. The main purpose of doing this is to utilize this natural passage of the umbilical cord to maximize the aesthetic appearance of the postoperative wound. Reduce the trauma of the child patients. Of course, this requires an experienced surgical team to operate. If the collection bag is damaged, there is a possibility of tumor implantation and metastasis. The umbilical incision was sutured, the abdomen was reinflated, and the laparoscope was reinserted to check the splenic wound for active bleeding. 7) A drainage tube was placed in the splenic fossa, fixed from the umbilical incision (**Figure 2I**), and the incision was sutured. There was approximately 30 ml of blood loss during the operation, and no transfusion was needed.

### 2.4 Patient postoperative conditions and pathological findings

**Figure 3:**
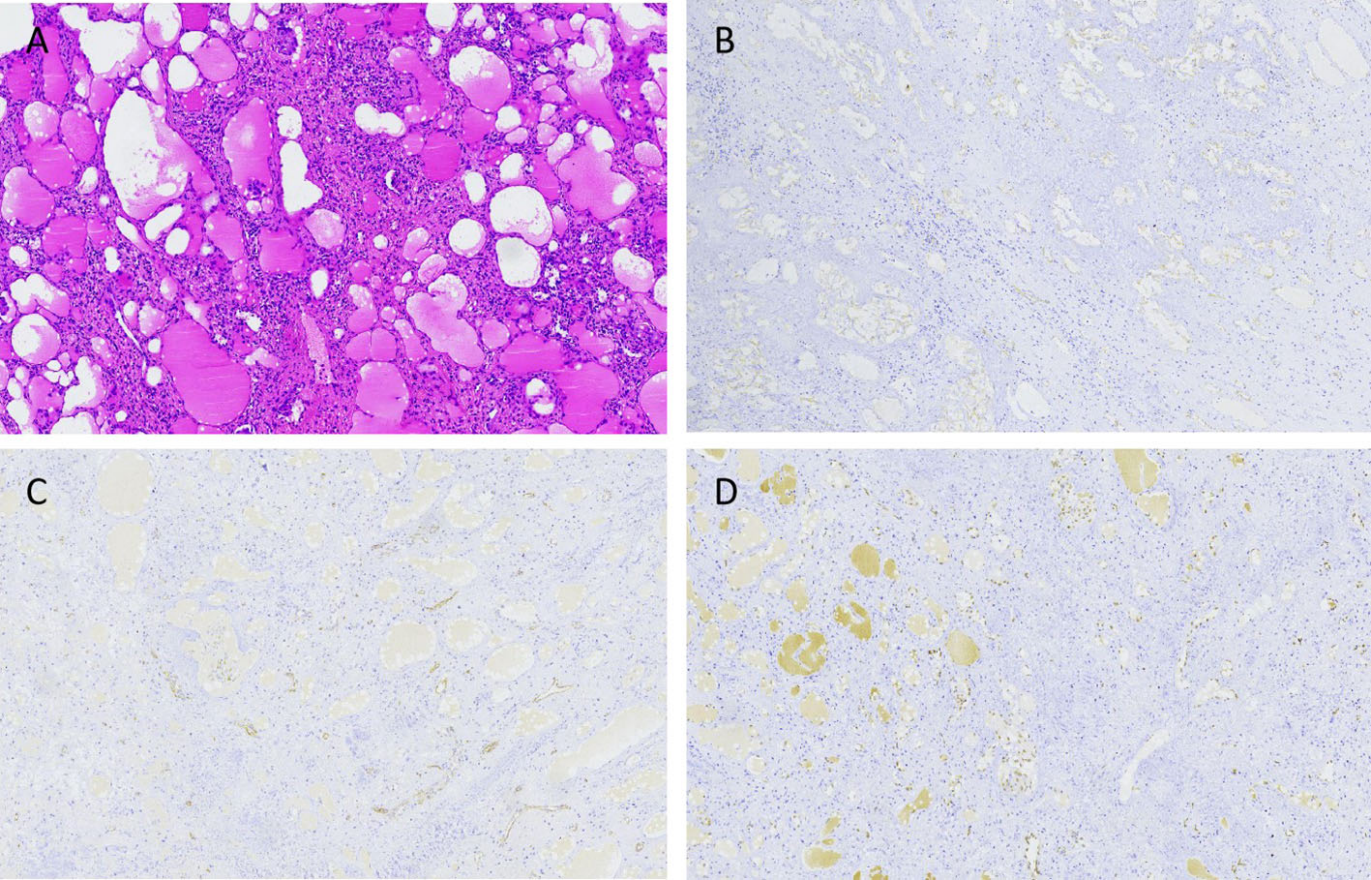
The hematoxylin-eosin (HE) and IHC results of the splenic PILA. **(A)** HE staining shows that the tumor is composed of numerous proliferating blood vessels, with papillary projections visible within the vascular lumen, endothelial cells appearing like a nail, and scattered lymphocyte infiltration around the blood vessels. No tumor components are seen at the tumor margin, consistent with intravascular papillary endothelial hyperplasia, ×40. **(B)** IHC results show positive expression of endothelial cells CD31, ×40. **(C)** IHC results show partial positive expression of CD34 in endothelial cells, ×40. **(D)** IHC results show positive expression of endothelial cells ERG, ×40.

**Figure 4:**
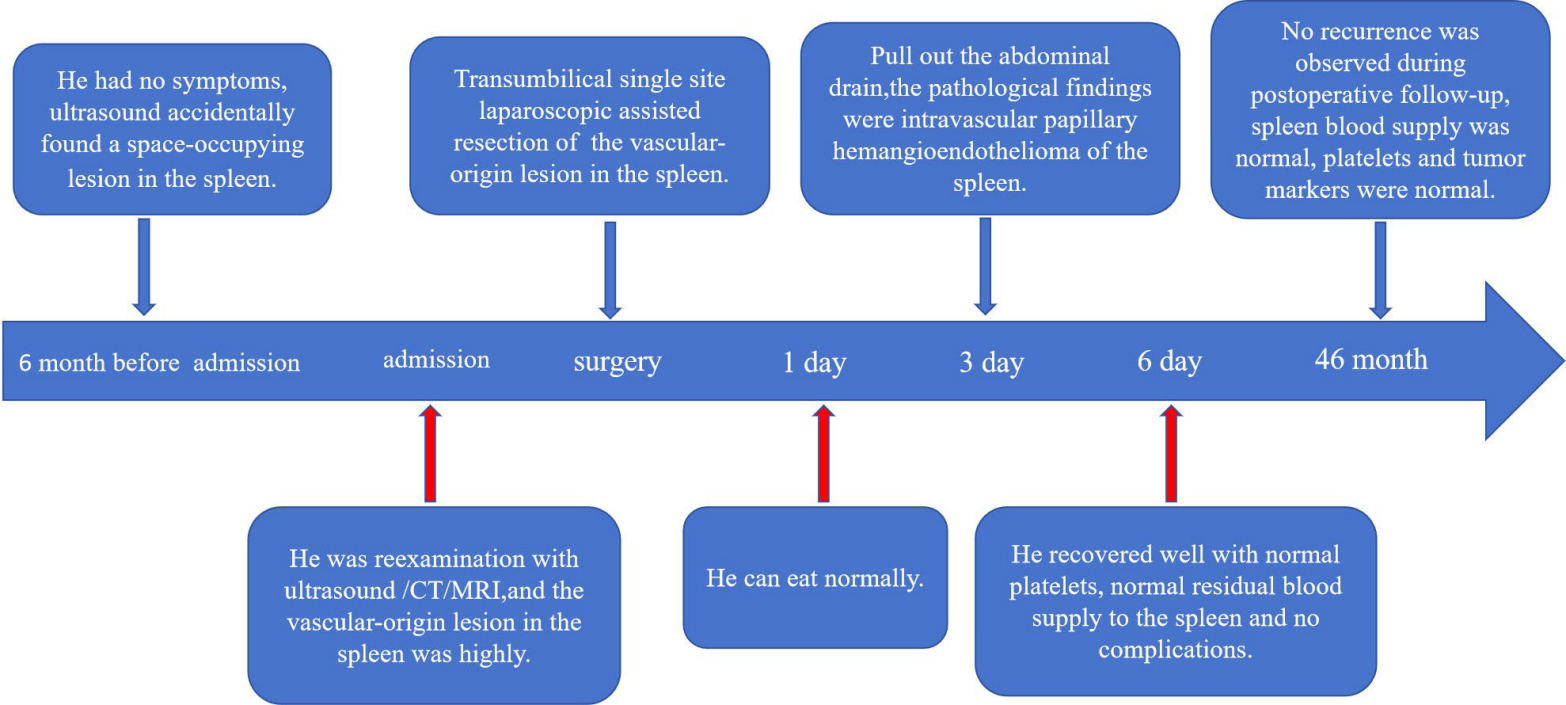
Complete timeline, including diagnosis, surgery, postoperative recovery, and follow-up.

The child started eating on the first postoperative day, the abdominal drainage tube was removed on the third day, and on the sixth day, a blood cell analysis revealed a red blood cell count of 4.66×10^12^/L and a platelet count of 324×10^9^/L. A follow-up ultrasound of the spleen did not reveal any issues. The child was discharged on the seventh day. During hospitalization, there were no complications, such as intra-abdominal bleeding, pancreatic fistula, splenic embolism, or splenic infarction. The excised cystic tissue visibly contained multiple irregular pieces of splenic tissue, with a gray–red surface and a central gray–yellow necrotic area on the cut surface. The pathological examination results revealed an intermediate vascular tumor consistent with intravascular papillary endothelial hyperplasia, with no tumor components observed at the margins (**Figure 3A**). Immunohistochemical (IHC) results revealed CD31 (+), CD34 (+), and ERG (+) expression (**Figures 3B–D**). Follow-up for 46 months postoperatively revealed no recurrence of the tumor on abdominal ultrasound; blood cell analysis revealed a red blood cell count of 5.2×10^12^/L and a platelet count of 285×10^9^/L; and serum tumor marker data did not reveal any issues (**Figure 4**).

## 3 Discussion

Splenic PILA is a rare borderline tumor that is quite uncommon in clinical practice and lacks specific early symptoms. Most affected children have no symptoms, but as the spleen enlarges, they may experience left upper abdominal pain, discomfort, or palpable abdominal masses, with only a few patients presenting with splenic rupture and hypovolemic shock. There is a lack of specific preoperative diagnostic methods for this condition, and diagnosis often relies on postoperative pathological examination, which may cause delays in diagnosis and treatment. In this case series, a patient was admitted after an incidental finding of a splenic mass during an ultrasound examination. We successfully removed the tumor through single-port laparoscopic surgery via the umbilicus, and the final pathological diagnosis was splenic PILA.

Abdominal US, CT, and MRI are commonly used as diagnostic methods for identifying splenic masses (10). Abdominal ultrasound might reveal an enlarged spleen with uneven echogenicity and clear demarcation from the surrounding splenic parenchyma, with some lesions exhibiting hypoechoic or anechoic areas. CT scans typically reveal single or multiple round or oval low-density areas with clear boundaries and relatively uniform density; early enhancement scans often reveal ring-like or spoke-like patterns at the tumor margins and septations, which gradually extend toward the center over time (11). MRI shows a low signal on T1-weighted images and a high signal on T2-weighted images, with clear boundaries and uniform density; after enhancement, the lesion contours are clearly defined, with early ring-like enhancement at the margins and septations, which gradually fills the central area with contrast over time, showing changes that are isodense in the delayed phase. In this case, the imaging findings were consistent with those of vascular tumors, allowing for early detection of the lesion and assisting in the assessment of tumor malignancy; however, the final diagnosis of splenic PILA still depended on pathological examination and IHC results. This underscores the importance of integrating imaging and pathological findings in the diagnostic process of splenic masses to avoid misdiagnosis and missed diagnosis (12).

The pathological features of splenic PILA include a tumor composed of proliferating capillaries, with most vascular lumens being underdeveloped and anastomoses present between vessels. Vascular endothelial cell differentiation may occur, with little to no atypia, and immunohistochemical staining commonly reveals positive expression of CD31, CD34, and ERG (13). Importantly, differentiating splenic vascular lesions, including intravascular papillary endothelial hyperplasia (Masson tumor), epithelioid hemangioendothelioma, angiosarcoma, lymphangioleiomyomatosis-like Kaposi sarcoma, and retinal angiosarcoma (14), is crucial. The typical feature of PILA is the presence of atypical columnar endothelial cells lining papillary structures. In the formation stage of Kaposi sarcoma, a similar composition may also be present. Angiosarcoma may exhibit focal morphological features similar to those of PILA; in a study of 80 patients with soft tissue angiosarcoma, 14% presented evidence of papillary lobular structures (15). However, angiosarcoma is characterized by endothelial atypia and diffuse growth outside vessels. Unlike PILA, Masson tumors contain visible intravascular thrombi, lack columnar endothelial cells, and have a single layer of endothelial cells surrounding a clear matrix nucleus (16). Epithelioid hemangioendothelioma is characterized by endothelial cells with an eosinophilic cytoplasm. Tumor cells are arranged as cords, trabeculae, or sheets within a mucoid or clear matrix and are distributed outside the vessels (17). Therefore, high vigilance and accurate judgment are required during diagnosis. The result of sending a small piece of tissue for rapid pathological examination during the surgery may be inaccurate and cannot replace the final pathological result. After team discussions, in future similar cases, if there is a preoperative suspicion of benignity, we can take multiple tissue samples from different sites and levels of the lesion for rapid pathology, which can increase the accuracy of the results. In our case, postoperative pathological discussion confirmed the diagnosis of an intermediate vascular tumor, which is consistent with descriptions of Dabska tumors in the literature (18).

The PILA is a borderline tumor with low invasiveness, rarely metastasizes, and generally has a good prognosis (7, 18). However, there is a possibility of malignant transformation, and the continuous growth of tumors poses a risk of rupture and bleeding. Therefore, complete surgical resection of the tumor is the best treatment option for splenic PILA (19); in principle, the spleen should be completely removed. However, owing to increasing concerns about postsplenectomy infections in children and adults and considering the young age of the patients in this group, their immune function is not fully developed, and the lesion is located far from the splenic hilum, along with rapid intraoperative pathology suggesting a high likelihood of a benign tumor, we opted for partial splenectomy. The patient recovered smoothly postoperatively. It is particularly important to emphasize that, there are reports in the literature that administering hemophilus influenzae, pneumococci, and meningococci vaccines along with antibiotics before splenectomy is essential for preventing the occurrence of fulminant infections (20, 21). We did not prophylactically use these vaccines and antibiotics before the surgery. Based on our past experience, if a total splenectomy is chosen, we routinely use antibiotics postoperatively to prevent pneumococcal infections. Since partial splenectomy can preserve some splenic function, we did not use antibiotics, and no related complications occurred postoperatively. Of course, this is inappropriate, and in future clinical cases, we will perform prophylactic vaccination with these vaccines and antibiotics before the surgery.

In our case series, we summarized the following surgical operations and experiences: 1) Due to the high cost and operational limitations of commercial single-port devices, we opted for three traditional 5 mm trocars to perform single-port surgery. 2) The three trocars entering the umbilicus can easily collide and increase surgical difficulty; we recommend adequately freeing subcutaneous tissue on both sides to increase the distance between the two trocars. Additionally, we arranged the observation and working trocars in a shallow-deep configuration to reduce interference from the laparoscope on the instruments and collisions at the ends of the trocars (22). 3) We selected smaller-end trocars and, when necessary, performed “scissor-like” cross operations with instruments in both hands to minimize interference between external instruments. 4) Our team found that in the early cases of laparoscopic partial splenectomy for umbilical cysts, it is advisable to place a gastric tube preoperatively to clear gastric residue during the procedure and to expand the surgical space by releasing the pneumoperitoneum (10). 5) If the cyst is large, intraoperatively, it may be necessary to puncture and drain the cystic fluid for testing to rule out parasitic infections and malignancies, while ensuring that the cystic fluid does not leak out during the puncture. 4) Using the greater curvature of the stomach for suspension can offer excellent anatomical exposure; importantly, a small amount of splenic tissue should be sent for rapid pathology during surgery. If it is a benign tumor, partial splenectomy can be performed; if it is malignant, total splenectomy should be performed to prevent recurrence and metastasis, as reports of lymph node and pulmonary metastases exist in the literature (15). Regular follow-up is crucial after surgery. 5) Careful dissection of the secondary splenic pedicle vessels is necessary during surgery while avoiding excessive dissection of the splenic ligament and monitoring the blood supply to the remaining spleen to prevent ischemic infarction of the residual spleen postoperatively. If there is significant bleeding at the cut surface, a tourniquet can be used to occlude the splenic pedicle vessels. The cut surface can generally achieve hemostasis through microwave coagulation, and hemostatic materials can be applied if necessary. If major bleeding occurs during the procedure or if it is difficult to preserve the spleen, timely conversion to open surgery is essential to ensure the safety of the child as the top priority. 6) Utilizing the gravitational effect of the spleen, the patient’s position can be continuously adjusted to facilitate placing the spleen into a specimen bag. The specimen bag is then raised to the umbilical incision, and the incision is longitudinally opened to enlarge the umbilical incision, using oval clamps or hemostatic clamps to fragment and remove the sample in stages. Care should be taken not to exert excessive force when the specimen bag is pulled upwards to avoid rupturing the bag, which could contaminate the abdominal cavity with the specimen and blood, affecting the surgical process. 7) The surgeon must have proficient laparoscopic skills, and the assistant’s requirements are also high; it is best to have a consistent surgical team, which is crucial for the success of the operation. 8) It is also very important to carefully read the CT results of the spleen before the operation, so as to better know the direction and branch of the blood vessels in the spleen.

This surgical approach has limitations due to the relatively restricted surgical field, difficulties in anatomical exposure, and limitations in instrument operation. Therefore, doctors lacking experience in single-port laparoscopic surgery should not blindly pursue aesthetic outcomes in surgical incisions and attempt such surgeries. Thus, individualized treatment plans are particularly critical when dealing with cases of splenic intravascular papillary endothelial hyperplasia. In this case, we chose single-port laparoscopic partial splenectomy via the umbilicus, which, although relatively less common in children, demonstrated good minimally invasive effectiveness and favourable treatment outcomes. The advantages of this method include rapid postoperative recovery and few complications, making it especially suitable for pediatric patients. It not only effectively removes the tumor but also reduces postoperative pain and hospital stay, improving the patient’s quality of life. Additionally, the application of rapid intraoperative pathology provides important evidence for surgical decision-making, increasing the timeliness and accuracy of surgical choices. This surgical method can effectively remove the tumor while maximizing the preservation of normal splenic tissue, thereby reducing the risk of postsplenectomy infections. Long-term follow-up after surgery is also crucial for timely monitoring of the risk of recurrence. In this case, the patient was followed up for 46 months postoperatively without recurrence of the tumor or complications such as splenic infarction.

Through a review of nearly 40 years of literature, we found very few literature reports on splenic PILA (**Table 1**) (19, 23–25). Most literature reports that PILA occur in the skin locations and subcutaneous tissue (26, 27), while PILA of the spleen are very rare. The age range for the occurrence of PILA is quite broad (5 to 33 years), primarily affecting children and young adults. Clinical presentations are variable and nonspecific, ranging from asymptomatic incidental findings on routine ultrasound to symptomatic manifestations, including early satiety with weight loss or palpable abdominal/left upper quadrant masses. Imaging modalities, mainly CT, occasionally magnetic Resonance Imaging or US, aid in identifying splenic lesions, suggesting further investigation. The definitive treatment method always includes splenectomy, performed through traditional open surgery (2 cases) or laparoscopic (2 cases), with total splenectomy being the common approach. Importantly, all three reported cases followed up (6-12 months) showed no evidence of recurrence after resection. These findings have significant clinical implications. First, they emphasize the highly variable and nonspecific presentations of this rare tumor, highlighting the importance of considering it in the differential diagnosis of splenic masses, especially in young patients. Second, the consistent reliance on imaging (US, CT, MRI) underscores its critical role in initial detection, although the final diagnosis remains histopathological. Third, the commonly used total splenectomy, achievable through both open and laparoscopic methods, indicates its effectiveness as the primary treatment for these early presentations. The absence of mid-term recurrence (up to 12 months) in the reported cases suggests a potentially favorable prognosis after complete resection. However, the number of cases is extremely limited, and the follow-up time is relatively short, necessitating caution. Long-term vigilance for recurrence remains essential, and larger, multicenter studies are needed to clearly define the biological behavior of the tumor, optimal management strategies, and true long-term outcomes.

**Table 1:** Literature review of the splenic PILA.

Author/year	Gender	Age/year	Symptom	Auxiliary examination	Singleor multiple	Laparoscopy or traditional open surgery	Total resection or partial resection	F/u(month) recurrency
Li et al/2023 (23)	Female	33	upper left quadrant mass	CT	Multiple	Traditional open surgery	Total resection	6 No
Wang et al/2022 (19)	Male	18	Accidental discovery by ultrasound	MRI	Multiple	Laparoscopy surgery	Total resection	6 No
Rodgers et al/2007 (24)	Female	6	Early satiety and weight loss	CT	Single	Traditional open surgery	Total resection	12 No
Katz et al/1998 (25)	Male	5	Abdominal mass	CT	Multiple	Laparoscopy surgery	Total resection	–

## 4 Conclusions

In conclusion, pediatric splenic PILA represents an exceptionally rare and diagnostically challenging entity, necessitating meticulous differentiation from other splenic vascular neoplasms. As a borderline tumor with documented potential for local invasion and low-grade metastasis, complete surgical excision coupled with vigilant long-term surveillance remains paramount to mitigate recurrence risk and optimize clinical outcomes. Transumbilical single-port laparoscopic partial splenectomy, as demonstrated in this case, has emerged as a minimally invasive and organ-preserving therapeutic strategy. This approach not only aligns with the imperative to safeguard pediatric immune function but also minimizes postoperative morbidity, underscoring its suitability for children. Future studies should further validate its efficacy in larger cohorts while refining the criteria for intraoperative decision-making and patient selection.

### 4.1 Patient’s perspective

Patient’s parents: “We are very grateful to the attending doctor for successfully healing our child’s splenic PILA through minimally invasive surgery, and it seems that regular US examinations are necessary for the child”.

## Data Availability

The original contributions presented in the study are included in the article/supplementary material. Further inquiries can be directed to the corresponding authors.
